# A Rare 46,X,t(Y;10)(q12;p14) Balanced Translocation in Non-Obstructive Azoospermic Patient with Elevated FSH and LH Levels

**DOI:** 10.1155/2023/6722623

**Published:** 2023-11-13

**Authors:** Kousar Jahan Syeeda Khursheed, Mohammed Rahman Kaleemullah, Annu Joseph, Mohammed Hasan Al Durazi, Moiz Bakhiet

**Affiliations:** ^1^Department of Molecular Medicine, Princess Al-Jawhara Centre for Molecular Medicine, Genetics & Inherited Disorders, College of Medicine and Medical Sciences, Arabian Gulf University, Manama, Bahrain; ^2^Department of Clinical Laboratory, Molecular Pathology Unit, Sultan Qaboos Comprehensive Cancer Care and Research Centre, SQU Street, Al Khoud, Oman; ^3^Consultant Genitourinary Surgeon, Al Khaleej Polyclinic, Road No. 2901, Block No. 329, Salmaniya, Manama, Bahrain

## Abstract

Structural chromosomal aberrations like translocations have been shown to cause spermatogenic failure. We report a rare 46,X,t(Y;10)(q12;p14) balanced translocation in an otherwise healthy non-obstructive azoospermic male with high follicle-stimulating hormone (26.65 IU/L) and high luteinizing hormone (13.58 IU/L). The patient was referred to us after clinical, hormonal, and histopathological investigations to identify chromosomal abnormalities by karyotyping and fluorescence in situ hybridization (FISH). Analysis of the banding pattern by karyotyping followed by FISH confirmed reciprocal translocation and identified the breakpoints at Yq heterochromatin (Yq12) and 10p14. Further molecular tests including AZF microdeletion assay were done, and the results, which showed no mutations in the analyzed genes, were provided by the referring doctor. Thus, our study points to the importance of conventional cytogenetic techniques in the preliminary evaluation of a genetic abnormality in cases of infertility and would help the patient make an informed decision before pursuing assisted reproductive technology.

## 1. Introduction

The prevalence of male infertility is estimated to be around 9–15% in the general male population [1]. Non-obstructive azoospermia (NOA) is considered to be among the most severe forms of male infertility, which is characterized by the absence of sperm in the ejaculate due to impaired spermatogenesis [2]. An elevated level of serum follicle-stimulating hormone (FSH) is considered to indicate abnormal spermatogenesis [3]. Luteinizing hormone (LH) acts on the Leydig cells in the testes to stimulate the production of testosterone. In a condition known as compensated Leydig cell failure, an increase in LH levels does not proportionately increase the levels of testosterone to support spermatogenesis effectively [4]. This can result in impaired sperm production. The assessment of testicular function by the measurement of serum gonadotropins like FSH and LH is a method to identify and manage NOA [5].

There could be a number of factors leading to NOA, both genetic and environmental, making it a heterogeneous disease. The presence or absence of multiple factors in a single individual makes the treatment of the condition more complicated. Since genetic anomalies have been detected in about 25% of azoospermic males [6], pinpointing the underlying genetic cause would help the patient choose the right type of assisted reproductive technology (ART) and appropriate genetic counseling.

Y/autosomal chromosome translocations are rare, and their frequency is estimated to be about 1/2000 in the general population [7]. Here, we present the cytogenetic analysis of a balanced (Y;10) translocation in an adult non-obstructive azoospermic male with high FSH and LH levels.

## 2. Case Presentation

Voluntary consent was obtained from the 34-year-old patient evaluated for infertility. He was phenotypically normal; clinical examination revealed a normal penis and regular testicular size and consistency. At least 4 sperm counts were attempted at approximately 4 month intervals, but mature spermatozoa could not be found.

Both epididymides were normal in the echo pattern under ultrasound examination. Scrotal color Doppler ultrasound (CDUS) study done on recumbent and erect positions with and without Valsalva maneuver indicated left varicocele with a caliper of 3.5 mm. Subsequent histopathology revealed dilated segments of veins with mural hyperplasia, which was consistent with bilateral varicocele. Left and right testicular biopsies identified the presence of early spermatids and the absence of mature spermatozoa in both samples, indicating germ cell maturation arrest with a Johnsen score of 7. With the above observations, the patient was diagnosed with non-obstructive azoospermia.

Reproductive hormone levels were within range for testosterone (9.80 nmol/L; normal range 9.1–40 nmol/L) and prolactin (8.40 ng/mL; normal range 2.7–11.0 ng/mL), while the levels of follicle-stimulating hormone (26.65 IU/L; normal range 1.6–11.0 IU/L) and luteinizing hormone (13.58 IU/L; normal range 0.8–6.0) were found to be elevated.

### 2.1. Karyotyping

**Figure 1:**
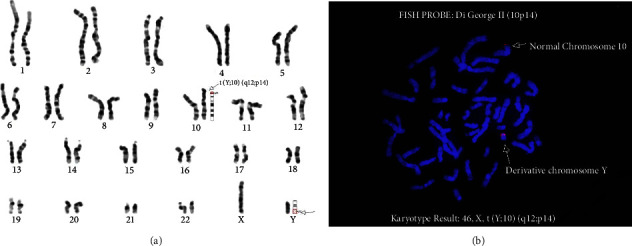
(a) Karyotype from metaphase chromosomes (GTG banding). Arrows indicate breakpoints. (b) Fluorescence in situ hybridization with DNA probes specific for Di George II gene *CUGBP2* (*CELF2*) at 10p14 (red).

The peripheral blood sample was collected from the patient and cultured in phytohemagglutinin- (PHA-) supplemented PB-MAX Karyotyping Medium (Thermo Fisher Scientific) for 72 hours. The cells were harvested, and the cell division was arrested at metaphase by treatment with 10 *µ*g/mL KaryoMAX Colcemid solution at a final concentration of 0.05 *µ*g/mL. After washing, the cells were subjected to hypotonic treatment with 0.075 mol/L KCl for 30 minutes at 37°C followed by fixation with Carnoy's Solution (3 : 1 ratio of methanol and acetic acid). Slides were prepared from the fixed cells and banded with Giemsa (GTG-banding). The chromosomes from 20 cells were karyotyped at a band resolution of 500 using CytoVision software (Leica Microsystems), and the karyotypes were interpreted according to ISCN 2020 criteria [8]. The result showed abnormal male karyotype 46,X,t(Y;10)(q12;p14) with a balanced translocation between the long arm of chromosome Y and the short arm of chromosome 10 (Figure 1(a)).

### 2.2. Fluorescence in Situ Hybridization (FISH) Analysis

From the fixed cell suspension, FISH analyses of 20 metaphase cells each from two sets of experiments were performed according to the manufacturer's protocol to confirm the karyotyping findings by using a probe. To confirm translocation from 10p14 to Yq12, we used a commercially available FISH probe (CytoCell Aquarius) specific for Di George II gene *CUGBP2* (*CELF2*) at 10p14 (Spectrum Red). Briefly, the probe was added to the slides prepared from fixed cells and kept for denaturation at 75°C for 2 minutes and hybridization at 37°C for overnight (ThermoBrite, Leica Biosystems). The slides were washed with 0.4x SSC at 73°C in a water bath for 2 minutes and 2x SSC with Tween 20 at room temperature for 30 seconds. They were then air-dried, DAPI was applied for 10 minutes, and images were taken using CytoVision software (Leica Microsystems).

The analysis confirmed the breakpoints of translocation which were earlier observed by karyotyping. The chromosome 10p14 region was observed on the long arm of chromosome Yq12, confirming the translocation (Figure 1(b)).

### 2.3. Mutation Studies in Candidate Genes for Infertility

Molecular screening to detect mutations in possible candidate genes for infertility was conducted, and the results were provided by the referring doctor. Sequence-tagged sites (STS) on AZFa, AZFb, and AZFc (AZF: azoospermia factor) regions as well as the heterochromatin region on the Y chromosome were analyzed by multiplex quantitative fluorescence-polymerase chain reaction (QF-PCR). The STS analyzed were sY84 and sY86 on AZFa, sY127 and sY134 on AZFb, sY254, and sY256 on AZFc, and sY160 on the heterochromatin region. Short tandem repeats (STR) on *SRY* and amelogenin genes were also amplified and analyzed. *SRY* markers, as well as the STS regions on AZF were all detected, and the dosage ratio of the amelogenin gene did not show a chromosome imbalance, indicating there are no microdeletions in the critical regions of the Y chromosome. Since 98% of individuals with a mutation in the cystic fibrosis transmembrane conductance regulator (*CFTR*) gene are known to have infertility issues [9], cystic fibrosis mutation screening was also done.

## 3. Discussion

High levels of FSH and LH have been suggested as a contributing factor of abnormality in spermatogenesis, a cause of NOA, by multiple studies [5, 10–12]. The study by Jahromi et al. has even suggested FSH plasma levels above 14.6 mIU/mL as a failure predictor of micro-TESE in NOA patients [5], while another study associated elevated FSH and LH with failed sperm retrieval [13]. Elevated FSH/LH levels combined with other factors such as varicocele and chromosomal abnormalities are indicators of hypergonadotropic hypogonadism/eugonadism [12]. The patient in our study was diagnosed with NOA with high FSH and LH by clinical and hormonal analyses.

Since chromosomal abnormalities are known to contribute to the disease condition in many cases, further tests were done to identify or eliminate the same. We could identify a Y;10 translocation in the patient from karyotype studies, which was confirmed by FISH analysis. Translocations involving Y and an autosome have been reported to affect germ cell maturation if the breakpoint is located within or near Yq11, where azoospermia factor AZFa is located [14].

Microdeletions in the AZF region have been reported in multiple studies in individuals with azoospermia. Since no microdeletions were detected in our molecular studies, the azoospermic condition of the patient is not a result of a deletion in the AZF region.

The location of the breakpoints is also another factor to be considered. If the breakpoint happened at the location of an active gene on the autosome or at the site of a regulatory factor of functional genes on the Y or autosome, it could hamper the production of specific proteins involved in spermatogenesis. This can only be confirmed by sequencing the breakpoint regions. A preliminary search in the UCSC Genome Browser on Human database (GRCh38/hg38) revealed four genes (*VAMP7*, *ILR9*, *WASH6P*, *SPRY3*) on Yq12, and 16 genes (*ITIH5*, *GATA3*, *USP6NL*, *UPF2*, *SFMBT2*, *ITIH2*, *DHTKD1*, *KIN*, *CELF2*, *ECHDC3*, *NUDT5*, *TAF3*, *PROSER2*, *ATP5F1C*, *SEC61A2*, and *CDC123*) on 10p14 [15]. While there have been no definitive reports on the direct effect of these genes on spermatogenesis, copy number variation of *VAMP7* has been associated with impaired male urogenital development [16]. Also, *Upf2*-knockout mice exhibited male sterility due to the depletion of Sertoli cells and germ cells during testicular development [17].

Although breakpoints at Yq11 are normally associated with infertility, there have been reports of translocations involving breakpoints at Yq12 pseudoautosomal region 2 (PAR2) on the Yq telomere where the affected individuals are infertile [18, 19]. A case of azoospermic male with (Y;10)(q12;p13) translocation has been previously reported, with meiotic arrest at metaphase I. At the same time, translocations involving 10p14 have been reported in males with oligozoospermia and miscarriages [20]. Reciprocal translocations between the Y chromosome and chromosome 10 which affect gonads or fertility reported in previous studies, to our knowledge, are shown in Table 1.

## 4. Conclusion

In conclusion, we report a 46,X,t(Y;10)(q12;p14) balanced translocation in an otherwise healthy patient with high FSH and high LH, who displayed spermatogenic arrest and hence NOA. Since the AZF region was also free from microdeletions, identifying whether the breakpoints occur at the site of active genes could also be important. This can be ascertained by sequencing the breakpoint regions.

The study highlights the importance of conventional karyotyping and FISH techniques to detect chromosomal abnormalities and genetic risk factors and would help with appropriate genetic counseling, as well as help the patient make an informed decision regarding the possibility of ART. The limitation of our study was that the actual molecular mechanism during meiosis and the DNA sequence at the breakpoints could not be elucidated under our resources and would need to be clarified by further molecular studies. Nevertheless, from our study, it is suggested that the patient should pursue assisted reproduction and possibly select female embryos by preimplantation genetic diagnosis (PGD).

## Figures and Tables

**Table 1:** Reciprocal translocations between Y chromosome and chromosome 10 reported in literature.

Reference	Karyotype	Conditions associated with gonads and fertility
Pajares IL, 1979	46,X,t(Y;10)(q12;p13)	Azoospermia, with arrest at metaphase I

Dallapiccola B, 1979	46,X,t(Y;10)(p11;p12)	Cryptorchidism and hypoplastic scrotum (28 months old with mental retardation)

Vialard F, 2009	46,X,t(Y;10)(q11.2;p15.2)	Fertile, with moderate asthenospermia and severe teratospermia

## Data Availability

All data are available upon request to the corresponding author.
